# Preventive Effect of *Chenopodium formosanum* Koidz. on Dextran Sulfate Sodium-Induced Chronic Colitis in Mice

**DOI:** 10.3390/nu18060959

**Published:** 2026-03-18

**Authors:** Hsing-Jung Yeh, Hung-Ming Chao, Chun-Chao Chang, Wei-Yu Kao, Suh-Ching Yang, Jane C.-J. Chao, Chun-Kuang Shih

**Affiliations:** 1 Division of Gastroenterology and Hepatology, Department of Internal Medicine, Taipei Medical University Hospital, Taipei 11031, Taiwan; yiew@ms10.hinet.net (H.-J.Y.); chunchao@tmu.edu.tw (C.-C.C.); 121021@h.tmu.edu.tw (W.-Y.K.); 2 School of Nutrition and Health Sciences, College of Nutrition, Taipei Medical University, Taipei 11031, Taiwan; jhaojhao0613@gmail.com (H.-M.C.); sokei@tmu.edu.tw (S.-C.Y.); chenjui@tmu.edu.tw (J.C.-J.C.); 3 Nutrition Research Center, Taipei Medical University Hospital, Taipei 11031, Taiwan; 4 TMU Research Center for Digestive Medicine, Taipei Medical University, Taipei 11031, Taiwan; 5 Division of Gastroenterology and Hepatology, Department of Internal Medicine, School of Medicine, College of Medicine, Taipei Medical University, Taipei 11031, Taiwan; 6 Division of General Medicine, School of Medicine, College of Medicine, Taipei Medical University; Taipei 11031, Taiwan; 7 Graduate Institute of Metabolism and Obesity Sciences, College of Nutrition, Taipei Medical University, Taipei 11031, Taiwan; 8 Taipei Cancer Center, Taipei Medical University, Taipei 11031, Taiwan; 9 Master Program in Global Health and Health Security, College of Public Health, Taipei Medical University, Taipei 11031, Taiwan; 10 School of Food Safety, College of Nutrition, Taipei Medical University, Taipei 11031, Taiwan

**Keywords:** *Chenopodium formosanum* Koidz., djulis, inflammatory bowel disease (IBD), dextran sulfate sodium (DSS), chronic colitis

## Abstract

**Background**: *Chenopodium formosanum* Koidz. (djulis) is an indigenous cereal crop native to Taiwan, and its effects on patients with inflammatory bowel disease (IBD) warrant exploration. The present study investigated whether the consumption of djulis can alleviate chronic colitis induced by dextran sulfate sodium (DSS) in mice. **Methods**: Forty mice were randomly divided into five groups: blank group (B), control group (C), low-dose group (L), medium-dose group (M), and high-dose group (H). Body weight and disease activity index (DAI) were recorded throughout this study. Groups C, L, M, and H were administered 2% DSS water on days 1–5 and 10–15 to induce chronic colitis. Groups L, M, and H were administered 5%, 10%, and 15% djulis, respectively. Serum and colon samples were collected for further analysis. **Results**: The DAI scores of groups L, M, and H were significantly lower than those of group C (*p* < 0.05), and the DAI scores of group H on day 18 were significantly lower than those of group L (*p* < 0.05). Colon length analysis revealed that DSS intervention significantly shortened colon length in group C (*p* < 0.05), whereas mice consuming djulis (groups L, M, and H) exhibited a restoration of colon length, with the effect being most pronounced in group H. DSS significantly increased the secretion of certain pro-inflammatory cytokines in the serum, such as interleukin (IL)-1β (*p* < 0.05), and the expression of some pro-inflammatory proteins in the colon, such as the nuclear factor kappa-light-chain-enhancer of activated B cells (NF-κB) (*p* < 0.05); however, djulis reversed these effects (especially in group H). In addition, mice in group H exhibited beneficial gut microbiota. **Conclusions**: Djulis alleviated chronic colitis in mice by reducing inflammation and modulating the gut microbiota. Further research is required to confirm these potential benefits in humans and elucidate the mechanisms involved.

## 1. Introduction

Inflammatory bowel disease (IBD) is a chronic inflammatory immune disorder characterized by recurrent digestive tract inflammation. IBD is categorized into two types: Crohn’s disease (CD) and ulcerative colitis (UC) [1]. Patients with IBD often experience symptoms such as abdominal pain and bloody stools, which adversely affect their quality of life. The etiology of IBD involves various factors, including diet, genetics, environment, microbiome, and immune dysregulation [2]. Among these, dietary nutrition and its role in IBD have garnered considerable attention [3]. Studies have indicated that the peak age of onset is 20–39 years for CD and 40–59 years for UC [4]. In the United States, the prevalence of IBD in adults increased from 0.9% in 1999 to 1.3% in 2015 [5]. In Taiwan, the incidence of UC has increased from 1.16 cases per 100,000 people in 2016 to 1.53 cases per 100,000 people in 2020, while that of CD has increased from 0.65 cases per 100,000 people in 2016 to 0.81 cases per 100,000 people in 2020 [6]. The rising prevalence of IBD in both Eastern and Western countries may be associated with the westernization of dietary habits [7]. Studies have indicated that the consumption of ultra-processed foods is associated with an increased risk of CD [8,9].

Various drugs are commonly used for IBD, including sulfasalazine, aminosalicylates, steroids, immunosuppressants, and biological agents [10,11]. Nutritional therapy is increasingly being recognized as a crucial aspect of IBD management. Adequate intake of dietary fiber and consumption of tea and coffee are considered protective factors [12]. A diet rich in fruits, vegetables, and *n*-3 fatty acids, along with low levels of *n*-6 fatty acids, may reduce the risk of CD and UC [3]. Patients with IBD are highly likely to develop avoidant or restrictive food intake disorders and sarcopenia owing to protein deficiency [3,13]. Additionally, the use of corticosteroids in patients with active IBD can exacerbate protein loss. Thus, it is recommended that these patients consume high-quality, protein-rich food, with a suggested protein supplement intake of 0.3 to 0.4 g/kg per meal [14,15]. Furthermore, diets high in inflammatory potential or those containing ultra-processed foods can increase the risk of IBD [8,16]. By contrast, dietary patterns or supplements that reduce inflammation may reduce the risk of IBD.

*Chenopodium formosanum* Koidz. (Djulis), a plant native to Taiwan and a traditional cereal of Taiwan’s indigenous people, is often consumed with rice or taro. Djulis seeds are rich in protein and dietary fiber and contain diverse essential amino acids such as lysine and histidine. Djulis contains twice the amount of lysine (the first limiting amino acid in cereals) found in corn and wheat, making it a cereal with high nutritional value [17]. Moreover, djulis is rich in carbohydrates, unsaturated fatty acids, minerals, and other nutrients, as well as in active compounds, such as rutin, kaempferol, quercetin, and betacyanins [17,18,19]. The predominant phytochemicals present in djulis are rutin (3.38 mg/g djulis) and betacyanins (2.36 mg/g djulis) [17]. These compounds can scavenge free radicals and reduce the expression of pro-inflammatory cytokines such as tumor necrosis factor-α (TNF-α) and interleukin (IL)-1β. Owing to the presence of these compounds, djulis exhibits antioxidant [20,21,22] and anti-inflammatory properties [23,24]. A previous study reported that djulis could improve liver fibrosis and liver damage [25]. Two experimental studies have demonstrated that djulis and its main compounds can ameliorate fatty liver induced by high-fat diets or liquid alcohol diets in obese mice [26,27], likely because of its role in inhibiting adipogenesis [18,28]. Quinoa grains, which are compositionally similar to djulis, contain various antioxidant and anti-inflammatory polyphenols [29,30,31]. Cereal polyphenols have beneficial effects on the gastrointestinal tract [32]. Some studies have reported that quinoa supports digestive health and alleviates colitis caused by dextran sulfate sodium (DSS) [33]. Given that djulis has a similar composition to quinoa but contains considerably higher levels of active ingredients (antioxidants and anti-inflammatory polyphenols) [34], djulis may have greater potential in preventing and alleviating colitis. However, there have been no studies showing the preventive effect and associated molecular mechanism of djulis on colitis. Moreover, djulis has been reported to inhibit colon cancer in animal models, and in cell models, djulis extract has been reported to inhibit the inflammatory response of colon cancer cells [35,36]. Lee’s articles also describe the phenolic [35] and nutritional composition [36] of djulis.

The present study used a mouse model to determine whether dietary djulis can prevent DSS-induced colitis by modulating the inflammatory response and gut microbiota.

## 2. Materials and Methods

### 2.1. Ethical Statements

The study and animal use protocols were approved by the Institutional Animal Care and Use Committee of Taipei Medical University (approval no. LAC-2021-0081) on 22 June 2021. The experimental animals were monitored once or twice per week for general health, including body weight, food and water intake, activity level, and posture, with additional observations if signs of distress were noted. Butorphanol was administered when animals exhibited signs of pain or distress. At the end of the experimental period, all animals were anesthetized with Zoletil^®^ (tiletamine–zolazepam, Virbac, Carros, France) combined with Rompun^®^ (xylazine, Dechra Pharmaceuticals, Northwich, UK) prior to terminal blood collection, in accordance with institutional guidelines. Humane endpoints were predefined, and animals meeting any of the following criteria were euthanized by carbon dioxide inhalation in consultation with a licensed veterinarian: unanticipated body weight loss exceeding 15%; inability to ambulate normally; inability to eat or drink; markedly reduced responsiveness to external stimuli; or persistent, pronounced vocalization over consecutive days.

### 2.2. Materials and Experimental Drugs

The djulis used in this study was purchased from Sinfong Agritech Co. (Taipei, Taiwan). Shelled djulis were ground into powder and stored at −20 °C until use [37]. Djulis powder was administered to mice as part of their diet. DSS is widely used to induce intestinal inflammation in mice and can effectively mimic the inflammatory response observed in IBD, particularly in UC [38,39,40]. The molecular weight of DSS ranges from 5 to 1400 kDa. DSS with a molecular weight of 36–50 kDa (MP Biomedicals, Santa Ana, CA, USA) is typically used to induce inflammation [39]. DSS forms nanovesicles by combining with medium-chain fatty acids in the intestine, allowing these vesicles to enter the body through the colon’s epithelial cells and trigger an inflammatory response [41]. The tissue characteristics and clinical symptoms of DSS-induced colitis are similar to those of UC [42]. As C57BL/6 mice are more sensitive to DSS, they are often used as the strain of choice for colitis models [43,44]. Forty 8-week-old male C57BL/6 mice were obtained from the National Laboratory Animal Center (Taipei, Taiwan). The mice were housed in well-maintained cages at a constant temperature of 21 °C and subjected to a controlled light–dark cycle. The experiments were designed in accordance with the recommended DSS dosage.

### 2.3. Study Design

**Figure 1:**
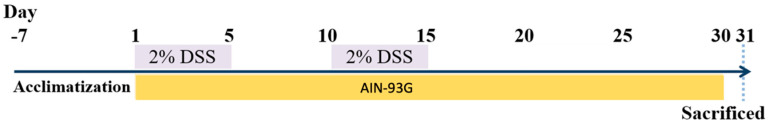
Experimental flowchart.

After a 1-week adaptation period, 40 8-week-old male C57BL/6 mice were randomly divided into five groups: blank group (B), control group (C), low-dose group (L), medium-dose group (M), and high-dose group (H; Table 1). Dietary intervention was initiated at the start of the experimental period (week 0). All groups were fed an AIN-93G-based standard diet. Groups B and D were fed the AIN-93G diet, while groups L, M, and H were fed the AIN-93G diet containing 5, 10, and 15% whole seed powder of djulis, respectively. The content of carbohydrates, fat, protein, and dietary fiber was adjusted according to the different amounts of djulis added, so the levels of macronutrients and dietary fiber in all groups were consistent. The proximate analysis of djulis and the composition of experimental diets are detailed in Supplementary Tables S1 and S2. The C, L, M, and H groups received 2% DSS in their drinking water to induce colitis on days 1–5 and 10–15, and normal drinking water was provided during the intervention periods. Body weight and food and water intake were monitored and recorded throughout the evaluation period. On the 31st day, the mice were sacrificed, and blood, colon, cecal content, liver, kidney, and spleen samples were collected for analysis. Figure 1 shows the experimental flowchart.

### 2.4. Measurement of Disease Activity Index

DSS-induced colitis in mice is characterized by clinical symptoms such as weight loss, diarrhea, and bloody stools [45]. The disease activity index (DAI) is commonly used to evaluate the severity of intestinal inflammation in animal models. The DAI scoring system includes assessments of changes in body weight, fecal consistency, and the presence of bloody stools, each of which is rated on a scale of 0 to 4 based on severity. The total score can reach 12 [46], with higher scores indicating more severe inflammation (Table 2).

### 2.5. Weighing of Organs

The liver, kidneys, and spleen of the mice were removed and rinsed with saline. After removing the excess water from the paper towels, the organs were weighed. In addition, the cecum content was collected, weighed, and stored in antifreeze tubes for subsequent microbiota analysis.

### 2.6. Histopathological Examination of the Colon

DSS-induced colitis can result in reduced colon length, mucosal epithelial cell damage, inflammatory cell infiltration, and mucosal atrophy [47]. In the present study, the colon of each mouse was removed and its length (in centimeters) was measured. A 0.5 cm section from the anal end was excised for histopathological analysis. The remaining colon was stored in antifreeze tubes for further analysis. The tissue section was stained with hematoxylin and eosin stain (H&E) and examined under a light microscope to evaluate the mucosal morphology and tissue changes for scoring (Table 3). Histopathological scoring was based on the structural integrity of the crypts (0–4 points), degree of inflammatory cell infiltration (0–3 points), and depth of tissue damage (0–3 points). The total score (maximum 10 points) indicated the severity of inflammation, with higher scores representing more severe inflammation [48]. Histopathological scoring was performed by a professional histopathology technician who was blinded to the experimental groupings.

### 2.7. Analysis of Inflammation-Related Cytokines and Proteins

Blood samples were collected from mice to enable the analysis of inflammation-related cytokines. The serum concentrations of TNF-α and IL-1β were measured using enzyme-linked immunosorbent assays (BioLegend, San Diego, CA, USA). Proteins were extracted from the colon tissue for analysis of inflammation-related proteins. Subsequently, Western blotting was performed to determine the expression levels of cyclooxygenase-2 (COX-2), nuclear factor kappa-light-chain-enhancer of activated B cells (NF-κB), and nuclear factor of kappa light polypeptide gene enhancer in B-cell inhibitor alpha (IkBα). COX-2 primary antibody was purchased from Abcam plc (Cambridge, UK). NF-κB primary antibody and IkBα primary antibody were purchased from Cell Signaling Technology, Inc. (Danvers, MA, USA). These protein levels were used to analyze the degree of colon inflammation [49].

### 2.8. Analysis of Gut Microbiota

Next-generation sequencing was performed to analyze the gut microbiota by Genomics Biotechnology, Inc. (New Taipei City, Taiwan). The mouse ceca were collected and homogenized. DNA was extracted from the samples using the QIAamp Fast DNA Stool Mini Kit (Qiagen, Hilden, Germany), in accordance with the manufacturer’s instructions. The V3-V4 high variation region of the 16S rRNA gene of the flora was amplified by polymerase chain reaction (PCR), using a specific primer set (341F: 5′-CCTACGGGNGGCWGCAG-3′, 806R: 5′- GACTACHVGGGTATCTAATCC -3′). PCR amplicons (Amplicon) were sequenced using Illumina MiSe (Illumina, San Diego, CA, USA). For each representative sequence, the feature-classifier and algorithm in QIIME2 were employed to annotate taxonomy classification based on the information retrieved from the Silva database. To normalize the variations in sequence depth across samples, amplicon sequence variant (ASVs) abundance information was rarefied to the minimum sequence depth using the QIIME script (single_rarefaction.py).

Quality control procedures were conducted at both experimental and bioinformatics levels. PCR products were verified by 1.5% agarose gel electrophoresis, and only samples showing a clear band at around 500 bp were selected for purification. Indexed libraries were further assessed using the Qubit 4.0 Fluorometer (Thermo Fisher Scientific, Waltham, MA, USA) and Qsep100 system (BiOptic, New Taipei City, Taiwan) prior to sequencing. For sequence data processing, raw reads were demultiplexed, and primer/adaptor sequences were removed using the QIIME2 cutadapt plugin. ASVs were generated using the QIIME2 DADA2 pipeline, which includes quality filtering, denoising, paired-end read merging, and chimera removal. Filtering was performed with a maximum expected error threshold of maxEE = 2 per read.

An operational taxonomic unit (OTU) was defined as a sequence sample with >97% similarity in its 16S rRNA gene sequence and was analyzed using QIIME analysis software QIMME2 version, while alpha as well as beta diversity was calculated using phyloseq to assess within- and between-group differences. The richness was analyzed by the linear discriminant analysis (LDA) effect size (LEfSe) method.

### 2.9. Statistical Analysis

Statistical analyses were performed using GraphPad Prism version 10.1.1 (GraphPad Inc., La Jolla, CA, USA). Data are presented as the mean ± standard deviation (SD). Differences between experimental groups were evaluated using Student’s *t*-test or one-way analysis of variance, followed by Tukey’s post hoc test. In the analysis of gut microbiota, differential abundance among groups was analyzed using the zero-inflated Gaussian model implemented in metagenomeSeq, with *p*-values adjusted using the false discovery rate (FDR). Differences in microbial community structures among groups were evaluated using the multi-response permutation procedure (MRPP), and *p*-values were corrected using the Benjamini–Hochberg adjustment. Statistical significance was set at *p* < 0.05.

## 3. Results

### 3.1. Body Weight, Food Intake, Water Consumption, and Food Efficiency in Mice

No significant differences were observed between the initial body weights of the mice in the B, C, L, M, and H groups. However, by the end of the study, group B had the highest final body weight among the five groups. The body weights of groups C, L, and M were significantly lower than those of group B. No significant difference in body weight was noted between groups H and B. Average weight gain was calculated by dividing the total weight gain by the number of days in the experimental period. The weight gain in group B was significantly higher than that in the other four groups. Water consumption was also significantly higher in group B than in the other four groups. In terms of food intake, no significant difference was observed between groups B and C. However, groups L, M, and H had significantly lower food intake than group B, and groups M and H had significantly lower food intake than group C. Feeding efficiency was calculated by dividing the weight gain by the food intake. The results revealed that group C had a significantly lower feeding efficiency than group B. Furthermore, no significant difference in feeding efficiency was observed between groups L and B (Table 4).

### 3.2. DAI

**Figure 2:**
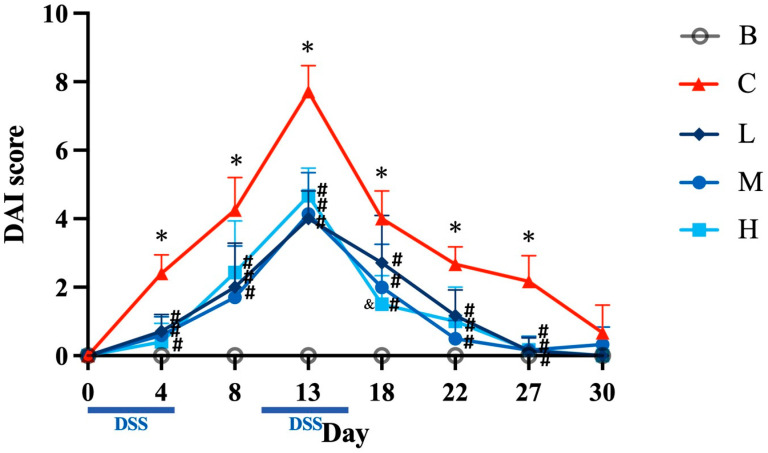
Effects of djulis on disease activity index (DAI) in DSS-induced chronic colitic mice. Data are presented as means ± SDs (*n* = 8). See Table 4 for details of the diet and experimental groups. Differences between experimental groups were evaluated using Student’s *t*-test. An asterisk (*) indicates a significant difference when compared with group B. A hashtag (#) indicates a significant difference when compared with group C. An ampersand (&) indicates a significant difference when compared with group L.

The results indicated that the DAI scores for group B did not increase throughout the experimental period. However, the DAI scores for the other groups peaked on the 13th day; this corresponded to the second cycle of DSS administration. The DAI scores on days 4, 8, 13, 18, 22, and 27 for groups L, M, and H were all significantly lower than those for group C. Furthermore, on day 18, the DAI score for group H was significantly lower than that for group L. By day 30, no significant differences in the DAI scores were observed among the five groups (Figure 2).

### 3.3. Weights of Liver, Kidney, and Spleen

**Figure 3:**
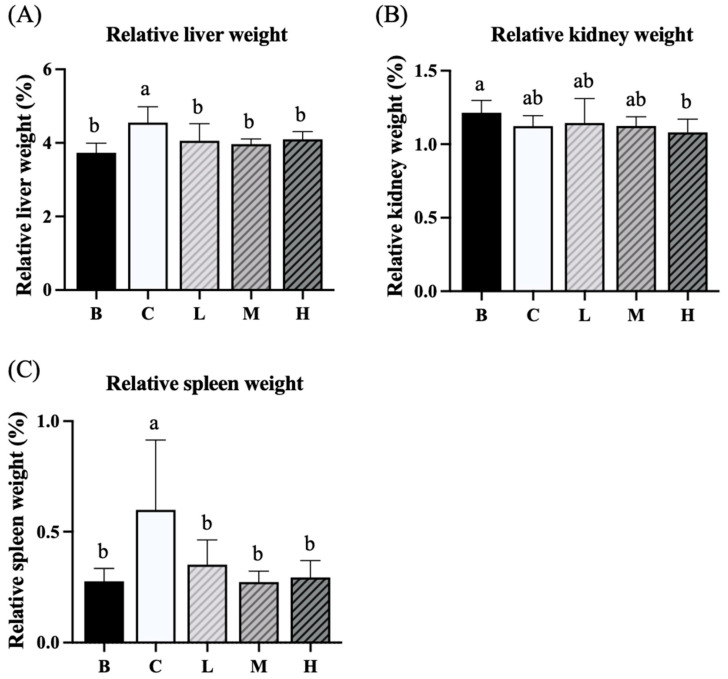
Effects of djulis on relative liver weight (**A**), relative kidney weight (**B**), and relative spleen weight (**C**) in DSS-induced chronic colitic mice. Data are presented as means ± SDs (*n* = 8). See Table 4 for details of the diet and experimental groups. Values with the same letter within a column are not significantly different from one another, as determined using ANOVA and Tukey’s post hoc test, *p* < 0.05.

The results demonstrated that the relative liver weights in group C were significantly higher than those in group B. However, groups L, M, and H had significantly lower liver weights than group C. Furthermore, the liver weights in groups L, M, and H did not differ significantly from those in group B (Figure 3A). Group H had a significantly lower relative kidney weight than group B, and no significant difference in the relative kidney weight was observed among the other groups (Figure 3B). This result indicates that high doses of djulis may affect kidney function. Furthermore, group C had a significantly higher relative spleen weight than that of group B. In addition, groups L, M, and H had significantly lower relative spleen weights than group C did. The relative spleen weights in groups L, M, and H did not differ significantly from those in group B (Figure 3C).

### 3.4. Weights of Cecum, Cecal Wall, and Cecal Contents

**Figure 4:**
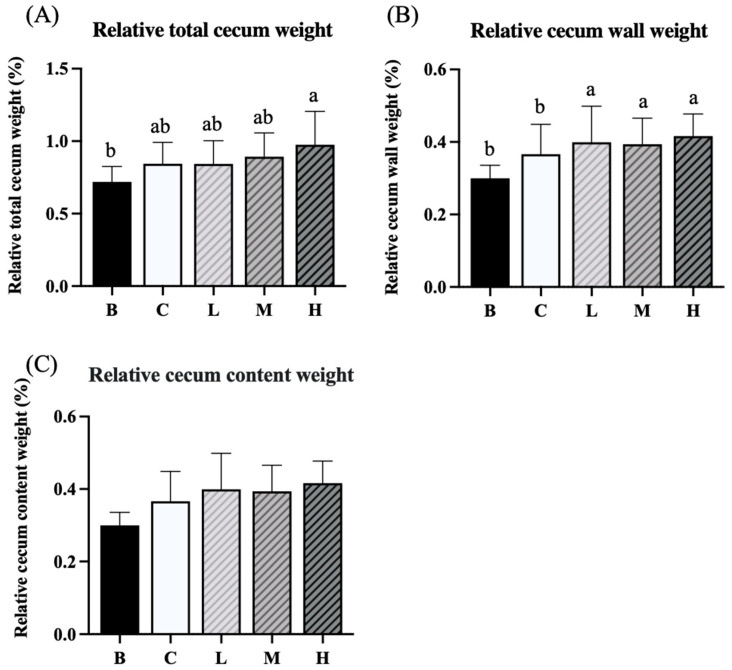
Effects of djulis on relative total cecum weight (**A**), relative cecum wall weight (**B**), and relative cecum content weight (**C**) in DSS-induced chronic colitic mice. Data are presented as means ± SDs (*n* = 8). See Table 4 for details of the diet and experimental groups. Values with the same letter within a column are not significantly different from one another, as determined using ANOVA and Tukey’s post hoc test, *p* < 0.05.

The experimental results indicated no significant difference in the relative total cecal weights between groups B and C, whereas group H had a significantly higher relative total cecal weight than group B (Figure 4A). The relative cecal wall weight did not differ significantly between groups B and C. However, groups L, M, and H had significantly higher relative cecal wall weights than group B (Figure 4B). Furthermore, no significant differences in relative cecal content were observed among the five groups.

### 3.5. Colon Length and Weight

**Figure 5:**
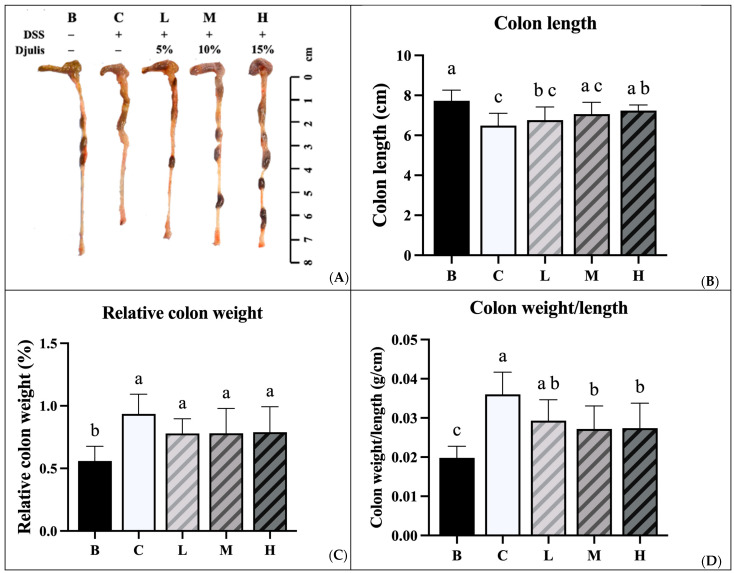
Effects of djulis on representative photographs of colon (**A**), colon length (**B**), relative colon weight (**C**), and colon weight/length ratio (**D**) in DSS-induced chronic colitic mice. Data are presented as means ± SDs (*n* = 8). See Table 4 for details of the diet and experimental groups. Values with the same letter within a column are not significantly different from one another, as determined using ANOVA and Tukey’s post hoc test, *p* < 0.05.

The experimental results indicated that the colon length of mice in group B was the longest of all the groups. Groups C and L had significantly shorter colon lengths than group B. However, no significant difference in colon length was observed between groups M and H and group B. Group H had significantly longer colon lengths than group C (Figure 5A,B). The relative colon weights of mice in groups C, L, M, and H were all significantly higher than those in group B (Figure 5C). We analyzed the ratio of colon weight to colon length and found that group B had the lowest ratio among the groups. In addition, groups M and H had significantly lower ratios than group C (Figure 5D).

### 3.6. Histopathological Evaluation of the Colon

**Figure 6:**
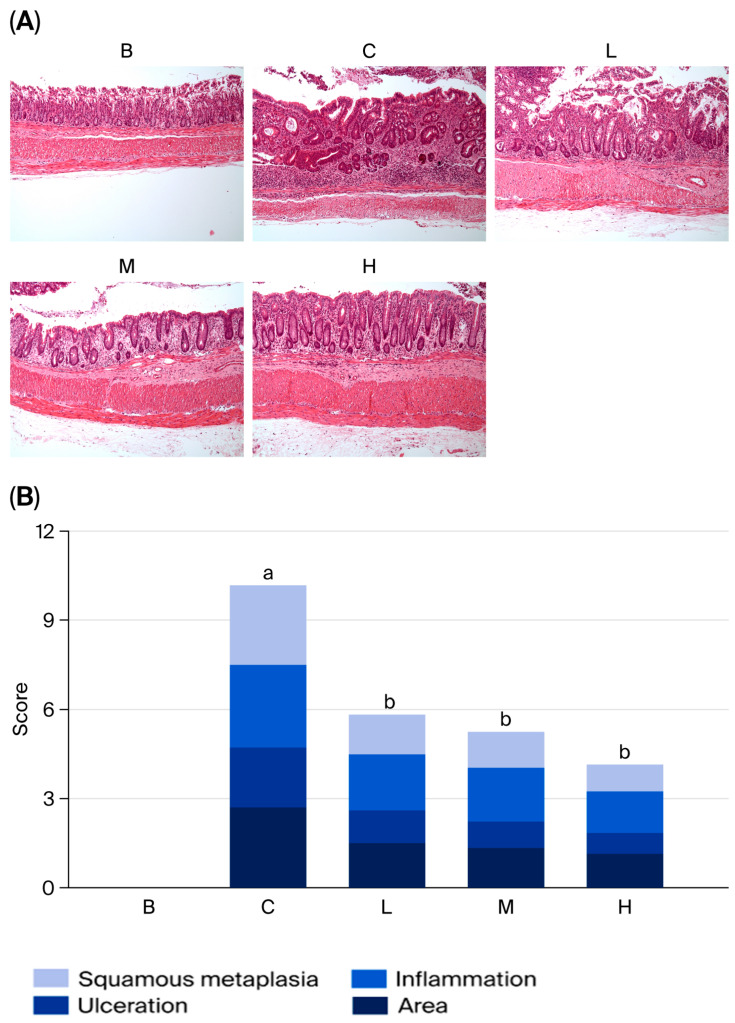
Effects of djulis on histopathological changes in DSS-induced chronic colitic mice. (**A**) H&E-stained sections of the colon (100×). (**B**) Histological score. Data are presented as means ± SDs (*n* = 8). See Table 4 for details of the diet and experimental groups. Values with the same letter within a column are not significantly different from one another, as determined using ANOVA and Tukey’s post hoc test, *p* < 0.05.

As presented in Figure 6A, after H&E staining of colon tissue sections, the colon structure in group B appeared intact, with crypts arranged in parallel columns and no immune cell infiltration. The histological score for this group was zero, indicating no pathology. In contrast, group C exhibited damaged crypt structures, reduced goblet cells, a disorganized arrangement, and immune cell infiltration. Group C had the highest histological score among the five groups, indicating that it had the most severe tissue damage. Groups L, M, and H exhibited better recovery than group C. These groups exhibited decreased immune cell infiltration, recovery of goblet cell development, and significantly lower histological scores than group C.

### 3.7. Analysis of Inflammatory Cytokines

**Figure 7:**
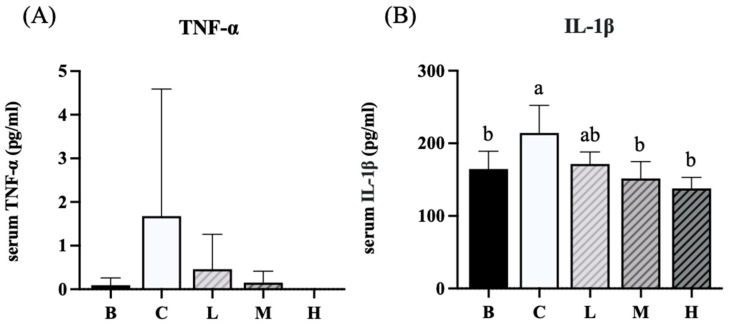
Effects of djulis on cytokines in DSS-induced chronic colitic mice. (**A**) TNF-α. (**B**) IL-1β. Data are presented as mean ± SDs (*n* = 8). See Table 4 for details of the diet and experimental groups. Values with the same letter within a column are not significantly different from one another, as determined using ANOVA and Tukey’s post hoc test, *p* < 0.05.

The levels of blood cytokines in each group are presented in Figure 7. Group C exhibited an increasing trend in TNF-α levels compared with group B and groups L, M, and H. However, this difference was not significant (Figure 7A). In contrast, the level of IL-1β in group C was significantly higher than that in group B. Groups M and H exhibited significantly decreased IL-1β levels compared to group C (Figure 7B).

### 3.8. Expression of Inflammation-Related Proteins

**Figure 8:**
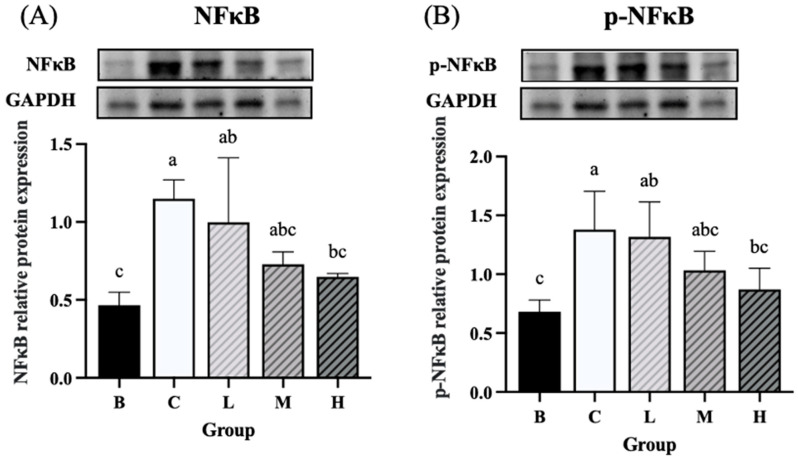
Effects of djulis on colon NF-κB and *p*-NF-κB protein expression in DSS-induced chronic colitic mice. (**A**) NF-κB. (**B**) *p*-NF-κB. Data are presented as means ± SDs (*n* = 3 to 4). See Table 4 for details of the diet and experimental groups. Values with the same letter within a column are not significantly different from one another, as determined using ANOVA and Tukey’s post hoc test, *p* < 0.05.

**Figure 9:**
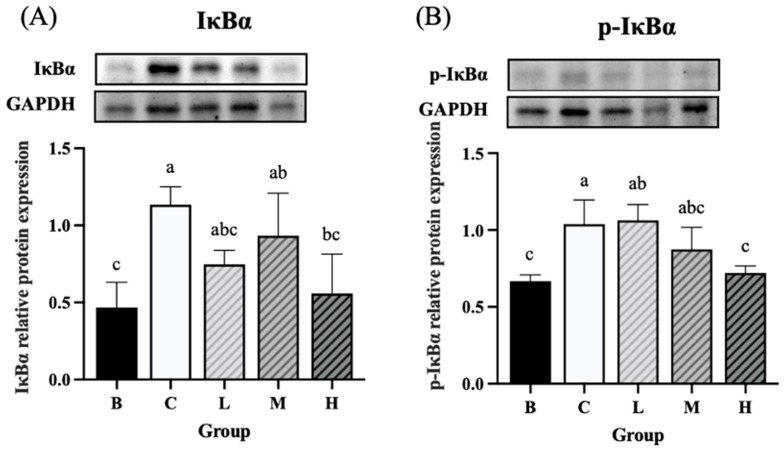
Effects of djulis on colon IκBα and *p*-IκBα protein expression in DSS-induced chronic colitic mice. (**A**) IκBα. (**B**) *p*-IκBα. Data are presented as means ± SDs (*n* = 3 to 4). See Table 4 for details of the diet and experimental groups. Values with the same letter within a column are not significantly different from one another, as determined using ANOVA and Tukey’s post hoc test, *p* < 0.05.

**Figure 10:**
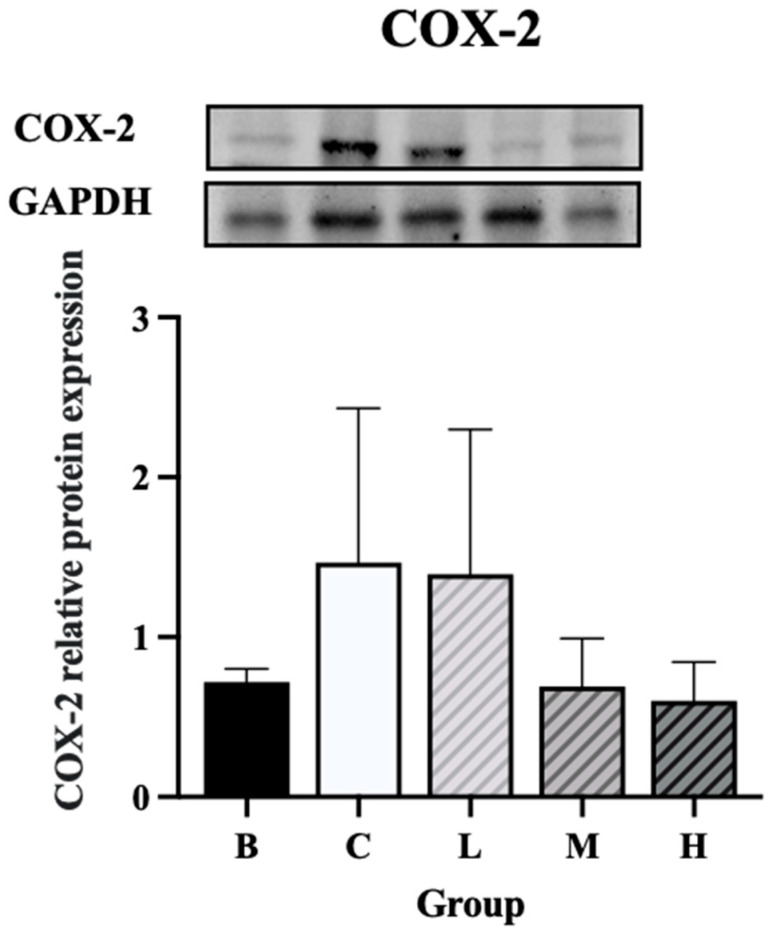
Effects of djulis on colon COX-2 protein expression in DSS-induced chronic colitic mice. Data are presented as means ± SDs (*n* = 3 to 4). See Table 4 for details of the diet and experimental groups.

The expression levels of NF-κB and *p*-NF-κB were significantly higher in groups C and L than in group B. On the other hand, group H had significantly lower expression levels of NF-κB and *p*-NF-κB than group C. Furthermore, the expression levels of NF-κB and *p*-NF-κB did not differ significantly between groups H and B (Figure 8A,B). The expression level of IκBα was significantly higher in groups C and M than in group B, whereas group H exhibited significantly lower levels of IκBα than group C. Furthermore, the level of IκBα did not differ significantly between groups H and B (Figure 9A). The expression level of *p*-IκBα was significantly higher in groups C and L than in group B. Group H exhibited a significantly lower expression level of *p*-IκBα than in group C. Furthermore, the level of *p*-IκBα did not significantly differ between group H and group B. Similarly, the expression level of *p*-IκBα in group M was not significantly different from that in group B (Figure 9B). The expression level of COX-2 tended to be higher in groups C and L than in group B, whereas groups M and H exhibited a decreasing trend in COX-2 expression compared to group C (Figure 10).

### 3.9. Gut Microbiota

**Figure 11:**
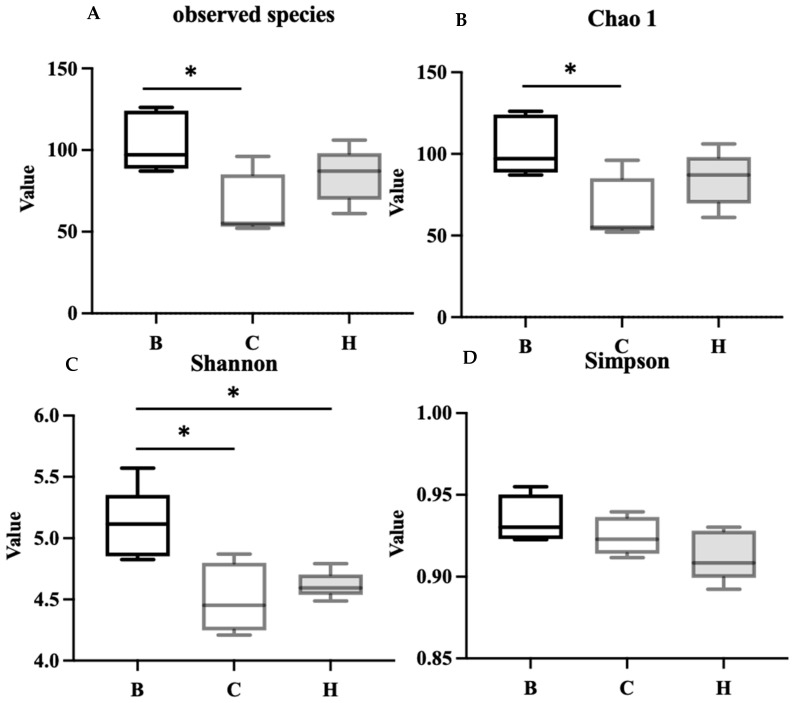
Effects of djulis on alpha diversity of microbiota in DSS-induced chronic colitic mice. (**A**) Observed species, (**B**) Chao1 index, (**C**) Shannon index, and (**D**) Simpson index. See Table 4 for details of the diet and experimental groups. Differences between experimental groups were evaluated using Student’s *t*-test. An asterisk (*) indicates a significant difference compared with group B.

As presented in Figure 11, the alpha diversity and richness of the bacterial communities were assessed using the observed species and Chao1, Shannon, and Simpson indices. The results indicated that group C had significantly lower observed species and Chao1 index values than group B, whereas group H exhibited no significant difference from group B, indicating a trend toward recovery. Group B had a higher Shannon index than groups C and H; however, no significant difference was noted between groups C and H. The Simpson index did not differ significantly among the three groups.

**Figure 12:**
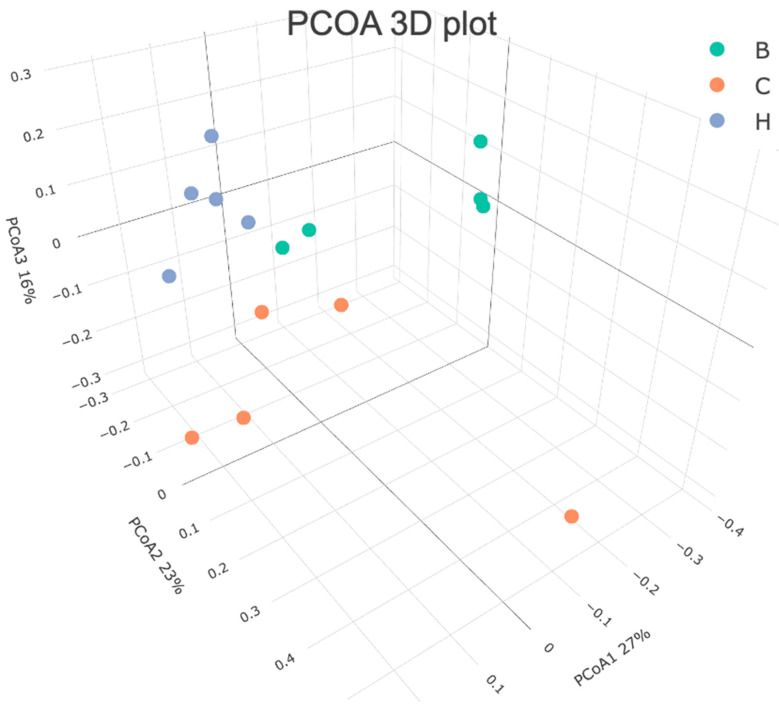
Effects of djulis on beta diversity of microbiota in DSS-induced chronic colitic mice. See Table 4 for details of the diet and experimental groups.

Figure 12 presents the results of the beta diversity analysis conducted using the principal coordinate analysis. The results demonstrated distinct separations in the bacterial communities among the three groups, with group H clustering closer to group B. ANOSIM analysis further confirmed significant differences in the bacterial community distributions among the three groups (Table 5).

**Figure 13:**
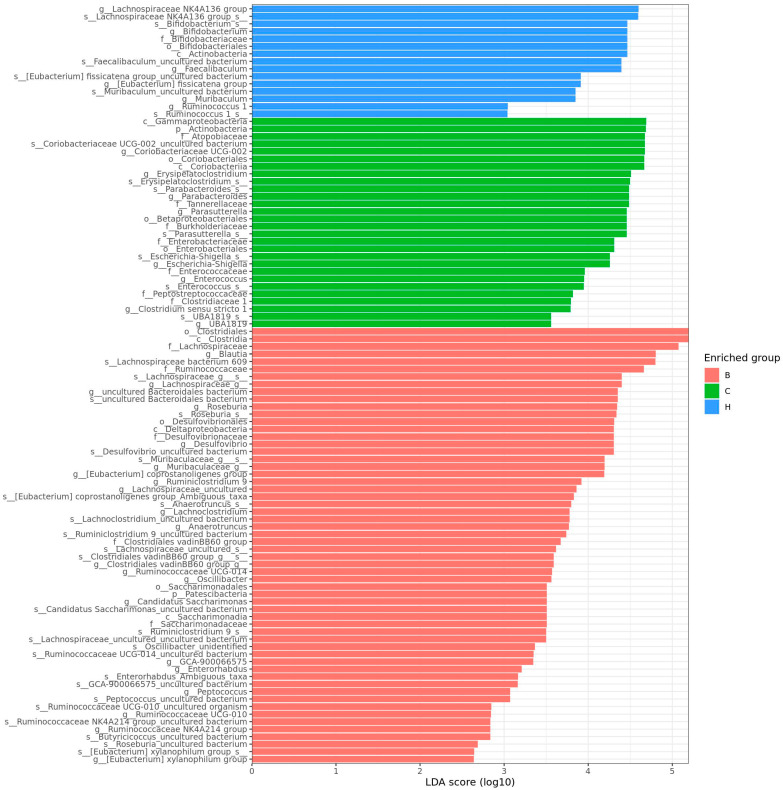
Effect of djulis on colonic microbial composition in DSS-induced chronic colitic mice. The length of the bar represents the LDA score and the colors indicate the group in which the taxa are more abundant compared with the other groups (*n* = 5). See Table 4 for details of the diet and experimental groups. Differentially abundant taxa are presented using the LEfSe method.

As depicted in Figure 13, the linear discriminant analysis (LDA) effect size score was used to identify the most representative bacteria in the intestinal flora. An LDA score greater than 2 indicated a significant difference in bacterial abundance among the groups. The analysis revealed that 58 species in group B were significantly different from those in the other two groups, whereas 28 species in group C and 15 species in group H showed significant differences.

**Figure 14:**
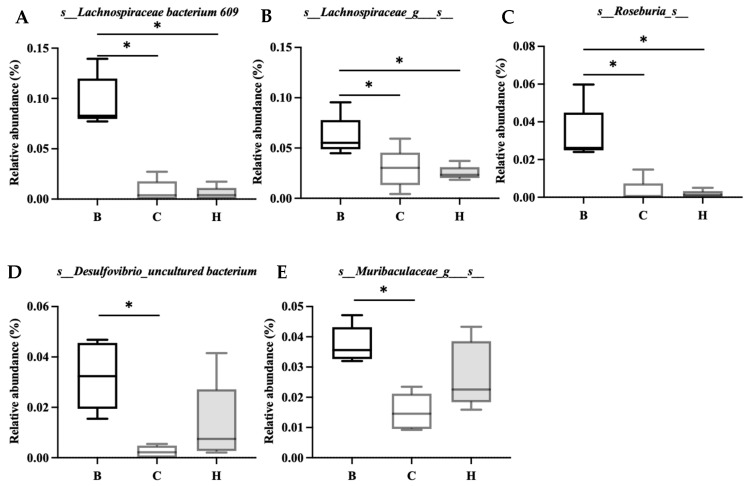
Effects of djulis on indicator species of microbiota in DSS-induced chronic colitic mice. (**A**) s_Lachnospiraceae bacterium 609, (**B**) s_Lachnospiraceae_g_s_, (**C**) s_Rosebuia_s_, (**D**) s_Desulfovibrio_uncultured bacterium, and (**E**) s_Muribaculaceae_g_s_. See Table 4 for details of the diet and experimental groups. Differences between experimental groups were evaluated using Student’s *t*-test. An asterisk (*) indicates a significant difference compared with group B.

Figure 14 lists the bacterial species that were significantly more abundant in group B, including Lachnospiraceae bacterium 609, Lachnospiraceae_g_s_, Roseburia_s_, Desulfovibrio_uncultured bacterium, and Muribaculaceae_g_s.

**Figure 15:**
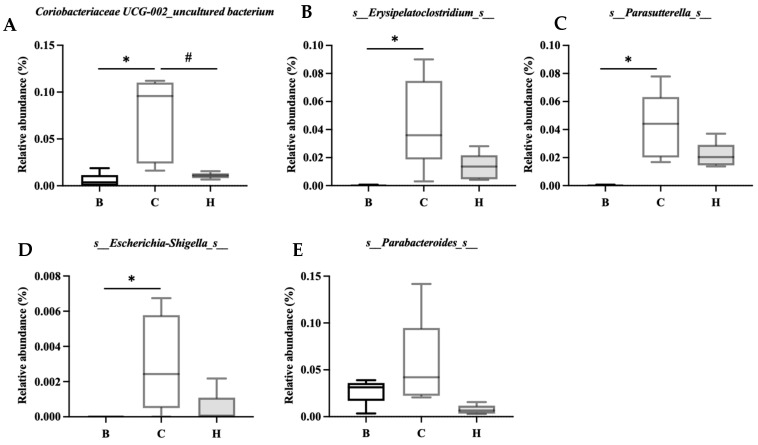
Effects of djulis on indicator species of microbiota in DSS-induced chronic colitic mice. (**A**) Coriobacteriaceae UCG-002_uncultured bacterium, (**B**) s_Erysipelatoclostridium_s__, (**C**) s_Parasutterella_s__, (**D**) s_Escherichia-Shigella_s_, and (**E**) s_Parabacteroides_s_. See Table 4 for details of the diet and experimental groups. Differences between experimental groups were evaluated using Student’s *t*-test. An asterisk (*) indicates a significant difference compared with group B. A hashtag (#) indicates a significant difference compared with group C.

**Figure 16:**
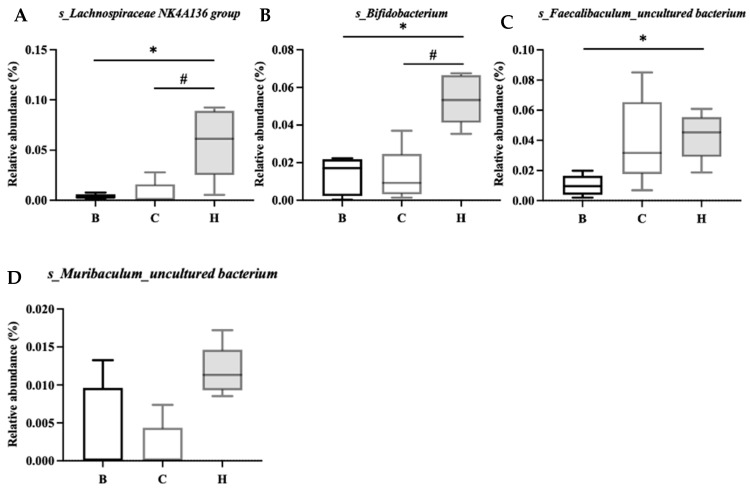
Effects of djulis on indicator species of microbiota in DSS-induced chronic colitic mice. (**A**) s_Lachnospiraceae NK4A136 group, (**B**) s_Bifidobacterium, (**C**) s_faecalibaculum_uncultured bacterium, and (**D**) s_Muribaculum_uncultured bacterium. See Table 4 for details of the diet and experimental groups. Differences between experimental groups were evaluated using Student’s *t*-test. An asterisk (*) indicates a significant difference compared with group B. A hashtag (#) indicates a significant difference compared with group C.

Figure 15 lists the bacterial species that were significantly more abundant in group C, namely, Coriobacteriaceae UCG-002_uncultured bacterium, Erysipelatoclostridium_s_, Parasutterella_s_, Escherichia-Shigella_s_, and Parabacteroides_s_. Figure 16 lists the bacterial species that were significantly more abundant in group H, which were primarily Lachnospiraceae NK4A136 group, Bifidobacterium_s_, Faecalibaculum_uncultured bacterium, and Muribaculum_uncultured bacterium.

## 4. Discussion

This study investigated the potential of whole grains to improve IBD conditions. A previous study conducted in Taiwan indicated that adopting a dietary pattern rich in whole foods might help prevent IBD or reduce its activity [50]. Djulis is rich in nutrients and exhibits anti-inflammatory properties. In this study, djulis was confirmed to have beneficial effects on colitis in mice. To our knowledge, this is the first study showing the preventive efficacy of djulis on colitis, and this may potentially contribute to the development of djulis as a novel health food product. One study investigated the effects of djulis hull extract [26], which is rich in rutin. In contrast, in the present study, we used whole grain djulis as feed for mice to examine its overall effect. The anti-inflammatory effects of the various parts of djulis warrant further investigation to understand their specific contributions and potential benefits.

The effects of consuming cereals such as djulis on COX-2, TNF-α, IL-1β, NF-kB, and IκBα in animals are worthy of discussion. An early study showed that COX-2 is involved in inflammation and promotes tumor formation and growth. COX-2 is rapidly induced by various extracellular and intracellular stimuli [51]. These stimuli include lipopolysaccharides (LPSs), interleukin-1 (IL-1), tumor necrosis factor (TNF), and arachidonic acid [51]. Another recent study found that under the stimulation of LPS and TNF-α, the secretion of IL-8 and the expression of COX-2 in HT-29 cells (human colon cancer cell line) increased significantly, showing a typical inflammatory response. The inhibition of cytokine IL-8 production can reduce the inflammatory response [52]. Whether the mechanism of the effect of djulis in reducing COX-2 is related to these mechanisms requires further study.

As a central regulator of the innate immune response of intestinal epithelial cells (IECs) and an important transcription factor that integrates the pro-inflammatory response to enteroinvasive bacterial infection, NF-κB can be activated by a variety of stimuli, such as TNF-α, IL-1, and LPS [53]. The transcriptional system in IECs plays an important role in regulating inflammation in patients with intestinal diseases [54]. It can regulate the transcription of genes related to the acute injury response and chronic intestinal inflammation, including genes related to factors such as IL-1, TNF-α, IL-6, IL-8, COX-2, and intercellular adhesion molecule -1 (ICAM-1) [52,55]. Another study showed that quinoa-derived peptides reduced NF-κB, IL-6, and IL-8 expression in colorectal cancer (CRC) cells by inhibiting histone deacetylase 1 (HDAC1) and inflammation [56]. This is similar to the results of another study that showed that rutin in djulis inhibited NF-κB activation, thereby reducing TNF-α and IL-1β levels and alleviating inflammation [25]. Djulis can reduce the degradation of IκBα, which is bound to NF-κB, and decrease the expression of pro-inflammatory cytokines [57]. Our study found that DSS increases the levels of NF-κB and *p*-NF-κB, but djulis decreases the levels of both. Therefore, one of the anti-inflammatory mechanisms of djulis may be related to its ability to inhibit the levels of NF-κB and *p*-NF-κB.

A systematic review of quinoa research showed that quinoa reduced the levels of TNF-α, IL-1β, and IL-6 in both animal studies and in vitro models [58]. Quinoa can reduce inflammation and thereby provide relief for inflammatory diseases, but its effect on systemic inflammation requires further research. The results of the systematic review are similar to the results of this study investigating the effects of djulis.

In addition, a study reported that a diet low in *n*-6 fatty acids and high in *n*-3 fatty acids could help reduce the risk of IBD [3]. However, djulis is rich in polyunsaturated fatty acids (PUFAs; 39.24%), with an *n*-6/*n*-3 ratio of 18.137 and a PUFA/saturated fatty acid ratio of 0.74 [59]. Although djulis contains high levels of *n*-6 fatty acids, its ability to reduce enteritis is not due to these fatty acids. Instead, the benefits of djulis may be associated with its high PUFA content, particularly the presence of long-chain *n*-3 PUFA, which can reduce the risk of UC [60]. Djulis also contains 24 bioactive compounds with multiple functions [28], such as increasing collagen production and reducing advanced glycation end-products and fatty oil droplets. Our previous study has found that whole djulis seeds are rich in phenolics and flavonoids, which help to regulate the antioxidant pathway in the colons of rats [35]. A substantial amount of evidence supports that phenolic compounds in quinoa and djulis have strong bioactivities, including antioxidation, anti-inflammation and intestinal homeostasis effects [19,31,61]. The antioxidant properties of djulis may alleviate intestinal inflammation in mice [28]. Compared to quinoa, djulis contains more antioxidants and anti-inflammatory polyphenols, which likely play a crucial role in the amelioration of DSS-induced colitis in mice [33,34].

Djulis may alleviate DSS-induced colitis in mice through its antioxidant, anti-inflammatory, and nutritive properties. Safety concerns regarding the excessive use of djulis exist, and the appropriate dosage for humans must be carefully studied. The maximum safe dosage of djulis should be determined before its use. Some studies have indicated that high doses of djulis can lead to an increase in glutamic pyruvic transaminase levels in rats. The estimated safe daily dose for adults is 126 g for shelled djulis or 70.5 g for unshelled djulis [62].

The intestinal tract contains a rich and diverse microbiome that includes bacteria, viruses, fungi, and protozoa, which constitute microbial flora [63]. The gut microbiota offers numerous health benefits to the host, including pathogen protection, nutritional support, metabolic improvements, and immune system enhancement. Various symbiotic interactions between the host and the microbiota are crucial for maintaining health [64]. The gut microbiota primarily comprises four bacterial phyla: Firmicutes, Bacteroidetes, Proteobacteria, and Actinobacteria [65]. Although a definitive relationship between specific bacteria and intestinal inflammation has not yet been established, studies have indicated that enteritis reduces the abundance and diversity of intestinal bacteria in mice. Enteritis mainly reduces the abundance of two major bacterial phyla, Firmicutes and Bacteroidetes, and increases the abundance of Proteobacteria. A substantial increase in the abundance of Proteobacteria is indicative of intestinal microbial dysbiosis [33,66].

IBD is associated with intestinal dysbiosis [67]. Whether or not nutritional supplements can improve intestinal flora imbalance, modify bacterial populations, and alleviate symptoms is currently an area of considerable research interest. The relationship between djulis and microbiota composition is also worth exploring. In the present study, among the mice with DSS-induced colitis, those fed high doses of djulis exhibited an increase in four bacterial species during recovery: s_Lachnospiraceae NK4A136, s_Bifidobacterium, s_Faecalibactulum_uncultured bacterium, and s_Muribaculum_uncultured bacterium. The Lachnospiraceae_NK4A136 group, which is considered to have anti-inflammatory properties, has been observed to increase in mice with reduced inflammation [68,69]. The genus Bifidobacterium has also been reported to ameliorate the symptoms of IBD [70,71], and Faecalibaculum has been reported to be associated with improvements in UC [72]. Although the abundance of Muribaculum was reported to typically decrease in mice with colitis, its abundance increased as inflammation improved [73]. The ability of djulis to enhance the presence of these four bacterial species in the intestine may indirectly improve intestinal dysbiosis.

Due to limited funding, this study only selected group H, which showed the best anti-colitis effect among all intervention groups, to analyze the gut microbiota. This selective analysis may limit dose–response interpretation and raise concerns about representativeness. However, group H remained the most representative intervention group and showed a significant effect in regulating the gut microbiota.

Although this study suggests that djulis may help improve DSS-induced colitis in mice, there are some limitations. First, this study lacked a positive control group, so we could not evaluate whether the changes relative to the DSS control group represent a modest modulation or an effect with potential protective implication. Without comparison to an established anti-colitis intervention, it is difficult to determine whether the observed improvements represent a modest modulation or a biologically meaningful protection. This substantially limits the translational relevance of the findings. Second, this study observed differences in food intake among groups, making it difficult to exclude the possibility that changes in caloric intake or nutrient composition had an impact on the reported outcomes. Even if diet formulations were theoretically matched, differential intake introduces a potential caloric and metabolic confounder. This factor may substantially weaken attribution of the observed physiological effects specifically to djulis itself. Third, the sample size of some experimental groups in this study was small, resulting in large variations in some data, so statistical difference might be obscured.

## 5. Conclusions

The findings of this study indicate that djulis exerts a protective effect against colitis in a DSS-induced mouse model. Colitis caused by DSS is similar to that caused by IBD, especially UC. Our results reveal that consuming djulis may help reduce inflammation and alleviate intestinal dysbiosis in mice. Mice being fed a diet containing 15% djulis is equivalent to a man weighing 60 kg consuming 112.8 g djulis per day. As a staple food, this is a feasible amount of djulis to consume. Djulis has the potential to be developed as a cereal product for gut health in the food industry. However, this is just a preliminary animal study, with a relatively small sample size and no positive control group. Therefore, further research is required to confirm these potential benefits in humans and fully elucidate the mechanisms involved.

## Figures and Tables

**Table 1:** Grouping and intervention for study mice.

Group	Diet	Intervention
B	None	Normal drinking water
DSS	None	2% DSS for 5 days and normal drinking water for 5 days (2 cycles)
LD	5% whole seed powder of djulis	
MD	10% whole seed powder of djulis	
HD	15% whole seed powder of djulis	

**Table 2:** DAI scores (Xiao et al., 2013) [46].

Score	Weight Loss	Stool Consistency	Fecal Occult Blood
0	None	Normal	Normal
1	<5%		
2	5–10%	Loose stools	Slight bleeding
3	10–15%		
4	>15%	Diarrhea	Gross bleeding

**Table 3:** Histopathological scoring (Bibi et al., 2018) [48].

Score	Crypt Damage	Severity of Inflammation	Depth of Tissue Damage
0	None	None	None
1	1/3 basal damage	Mucosal infiltration	Mucosal damage
2	2/3 basal damage	Mucosal and submucosal infiltration	Mucosal and submucosal damage
3	Only surface epithelium intact	Transmural infiltration	Transmural damage
4	Complete loss of crypt and epithelium		

**Table 4:** Effects of djulis on body weight, food intake, food efficiency, and water consumption in DSS-induced chronic colitis mice ^1,2^.

Group ^3^	Initial Body Weight (g)	Final Body Weight (g)	Weight Gain (g/d)	Water Consumption (g/d)	Food Intake (g/d)	Food Efficiency ^4^ (%)
B	23.6 ± 1.7	27.7 ± 1.6 ^a^	0.14 ± 0.02 ^a^	5.7 ± 1.0 ^a^	5.1 ± 0.7 ^a^	2.7 ± 0.5 ^a^
C	23.7 ± 1.4	25.0 ± 2.0 ^b^	0.04 ± 0.07 ^b^	4.7 ± 0.6 ^b^	4.7 ± 0.5 ^ab^	0.9 ± 1.4 ^b^
L	23.1 ± 1.4	25.2 ± 2.2 ^b^	0.07 ± 0.05 ^b^	4.0 ± 0.4 ^b^	4.3 ± 0.5 ^bc^	1.6 ± 1.2 ^ab^
M	23.4 ± 1.8	24.9 ± 2.1 ^b^	0.05 ± 0.02 ^b^	4.4 ± 0.8 ^b^	4.0 ± 0.6 ^c^	1.3 ± 0.5 ^b^
H	23.9 ± 1.1	25.3 ± 1.9 ^ab^	0.05 ± 0.04 ^b^	4.6 ± 0.5 ^b^	3.9 ± 0.3 ^c^	1.2 ± 1.1 ^b^

^1^ All values are presented as means ± SDs (*n* = 8). ^2^ Values with the same letter within a column are not significantly different from one another, as determined using ANOVA and Tukey’s post hoc test (*p* < 0.05). ^3^ All groups, except for group B, were administered DSS. Groups B and C: AIN-93G diet; group L: AIN-93G diet containing 5% djulis; group M: AIN-93G diet containing 10% djulis; group H: AIN-93G diet containing 15% djulis. ^4^ Weight gain/food intake × 100%.

**Table 5:** Results of ANOSIM ^1,2^.

Comparison	R Statistic Value	*p* Value ^3^	N. Perm
B vs. C	0.636	0.009	10,000
B vs. H	0.784	0.008	10,000
C vs. H	0.524	0.008	10,000

^1^ ANOSIM: analysis of similarities. ^2^ All groups, except for group B, were administered DSS. Groups B and C: AIN-93G diet; group H: AIN-93G diet containing 15% djulis. ^3^ *p* < 0.05 was considered significant.

## Data Availability

The experiments in this study were conducted under the leadership of the corresponding author and in accordance with the ethical guidelines of Taipei Medical University. The original contributions presented in this study are included in the article/Supplementary Materials. Further inquiries can be directed to the corresponding author.

## Supplementary Materials

The following supporting information can be downloaded at https://www.mdpi.com/article/10.3390/nu18060959/s1, Table S1. Proximate analysis of djulis (wet basis). Table S2. Composition of experimental diets.

## Abbreviations

The following abbreviations are used in this manuscript:

IBD	Inflammatory bowel disease
DSS	Dextran sulfate sodium
DAI	Disease activity index
CD	Crohn’s disease
UC	Ulcerative colitis
TNF-α	Tumor necrosis factor-α
IL	Interleukin
LPS	Lipopolysaccharide
H&E stain	Hematoxylin and eosin stain
COX-2	Cyclooxygenase-2
NF-κB	Nuclear factor kappa-light-chain-enhancer of activated B cells
IkBα	Nuclear factor of kappa light polypeptide gene enhancer in B-cell inhibitor alpha
SDs	Standard deviations score
SAS	Statistical Analysis System (version 9.4)
IECs	Intestinal epithelial cells
ICAM-1	Intercellular adhesion molecule -1
CRC	Colorectal cancer
HDAC1	Histone deacetylase 1
PUFAs	Polyunsaturated fatty acids
TNBS	Trinitrobenzene sulfonic acid
