# Supplementary material for: Preventive Effect of Chenopodium formosanum Koidz. on Dextran Sulfate Sodium-Induced Chronic Colitis in Mice
Source: Nutrients. 2026 Mar 18;18(6):959. doi: 10.3390/nu18060959 (PMC13029533; doi:10.3390/nu18060959)
Supplement: Supplementary file 1 [file nutrients-18-00959-s001.zip › nutrients-4131923-supplementary.pdf]

Supplementary Table S1. Proximate analysis of djulis (wet basis)

Composition	Content (g/100g)
Moisture	15.52
Ash	6.42
Crude fat	4.23
Crude protein	14.09
Total dietary fiber	23.30
Nitrogen-free extract[1]	36.44

[1]Nitrogen-free extract = 100 – moisture – ash – crude fat – crude protein – total dietary fiber.

Supplementary Table S2. Composition of experimental diets

Composition	Content[1] (g/kg diet)				
	B	C	L	M	H
Djulis			50	100	150
Corn starch	397.50	397.50	379.28	361.06	342.84
Casein	200	200	192.96	185.91	178.87
Sucrose	100	100	100	100	100
Dextrinized corn starch	132	132	132	132	132
Soybean oil	70	70	67.89	65.77	63.66
α-Cellulose	50	50	38.35	26.70	15.05
Mineral mix	35	35	35	35	35
Vitamin mix	10	10	10	10	10
L-Cystine	3	3	3	3	3
Choline bitartrate	2.5	2.5	2.5	2.5	2.5
tert-Butylhydroquinone (TBHQ)	0.01	0.01	0.01	0.01	0.01

[1]Groups B and C: AIN-93G diet; group L: AIN-93G diet containing 5% djulis; group M: AIN-93G diet containing 10% djulis; group H: AIN-93G diet containing 15% djulis.
